# Renal allograft interstitial fibrosis: multicellular interactions and therapeutic strategies

**DOI:** 10.3389/fimmu.2026.1745244

**Published:** 2026-02-04

**Authors:** Runmin Ding, Qinghuan Shen, Junyi Zhou, Ruoyun Tan, Min Gu, Zijie Wang, Zeping Gui

**Affiliations:** 1 Department of Urology, Jiangsu Key Laboratory of Urological Disease Prevention and Treatment, The Second Affiliated Hospital of Nanjing Medical University, Nanjing Medical University, Nanjing, China; 2 The Second Clinical Medical College, Nanjing Medical University, Nanjing, China; 3 Department of Urology, The First Affiliated Hospital with Nanjing Medical University, Nanjing, China

**Keywords:** chronic allograft dysfunction (CAD), immune cells, kidney transplantation, multicellular crosstalk, renal allograft interstitial fibrosis, renal cells

## Abstract

Kidney transplantation remains the most effective treatment for end-stage renal disease (ESRD). However, long-term graft survival is still limited by chronic allograft dysfunction (CAD), which is primarily characterized by renal interstitial fibrosis (RIF). The development of RIF is an actively regulated and progressive process involving both immune and non-immune mechanisms. Within the renal microenvironment, multiple cell populations interact to form a self-reinforcing profibrotic network that ultimately drives irreversible fibrotic remodeling. Despite increasing mechanistic insights, the precise modes of multicellular crosstalk remain incompletely understood, and effective targeted therapies are still lacking in clinical practice. In this review, we systematically summarize the dynamic interactions among immune cells, renal epithelial cells, and stromal cells during renal allograft interstitial fibrosis. By integrating recent advances at the cellular and molecular levels, we identify key regulatory nodes within this multicellular network and discuss emerging therapeutic targets and precision intervention strategies aimed at inhibiting profibrotic signaling, alleviating pathological tissue remodeling, and improving long-term graft function and survival.

## 1 Introduction

Renal transplantation remains the most effective treatment option for patients with end-stage renal disease (ESRD), offering significant advantages in prolonging survival and improving quality of life compared to dialysis therapy (1). Despite substantial improvements in short-term graft survival owing to advances in immunosuppressive regimens and surgical techniques, long-term outcomes remain suboptimal, with approximately 40–50% of grafts failing within 10–15 years post-transplantation due to chronic allograft dysfunction (CAD) (2, 3). Histologically, CAD is characterized by progressive glomerulosclerosis, tubular atrophy, and persistent inflammation, among which renal interstitial fibrosis (RIF) is recognized as the final common pathway leading to irreversible graft failure (4).

RIF is not merely a passive structural consequence of chronic injury but rather an actively regulated pathological process. It is characterized by excessive extracellular matrix (ECM) deposition, sustained immune activation, and aberrant tissue remodeling (5). Multiple injurious stimuli contribute to transplant-associated fibrosis, including acute and chronic rejection, ischemia-reperfusion injury (IRI), and the nephrotoxic effects of calcineurin inhibitors (6). Among these factors, injury to the peritubular capillary (PTC) network represents a critical initiating event. PTC damage reflects microcirculatory dysfunction and plays a central role in triggering the development and progression of RIF (7).

Importantly, limited fibrosis may help maintain renal architecture and function; however, once a positive feedback loop is established, ECM accumulation becomes excessive and disrupts nephron function, ultimately driving chronic renal failure (8). A hallmark of RIF at the cellular level is the accumulation of activated myofibroblasts, the primary effector cells responsible for ECM production (9). These myofibroblasts are derived from various sources, including resident fibroblasts, epithelial cells, endothelial cells, pericytes, and bone marrow-derived macrophages (10). The altered post-transplant microenvironment can also induce tubular epithelial cell cycle arrest, apoptosis, senescence, and transdifferentiation into myofibroblasts, further contributing to tubular atrophy and architectural damage (11). Notably, interstitial fibrosis and tubular atrophy (IF/TA) frequently coexist and are regarded as the most prominent histological features of chronic rejection, serving as key predictors of long-term graft outcomes (12).

Within the fibrotic niche, immune cells, renal epithelial cells, and stromal cells engage in complex intercellular crosstalk, orchestrated through profibrotic signaling hubs such as the TGF-β/Smad, IL-6/STAT3, and Hippo-YAP pathways (13, 14). These cascades interact through reciprocal feedback mechanisms that sustain inflammation and ECM deposition (15, 16). Beyond these classical pathways, emerging evidence highlights the role of mitochondrial dysfunction, lipid metabolic reprogramming, and epigenetic dysregulation in promoting fibrogenesis, offering novel avenues for therapeutic targeting (17, 18). Moreover, soluble inflammatory mediators released within the fibrotic microenvironment may further amplify immune responses, establishing a vicious cycle of inflammation and fibrosis (12).

In summary, renal transplant-associated fibrosis is a multifactorial and multicellular pathological process governed by complex and interwoven signaling networks. A deeper understanding of the underlying mechanisms, including key cellular interactions and regulatory pathways, is crucial for identifying targeted antifibrotic strategies and ultimately improving long-term graft survival and patient prognosis.

## 2 Role of immune cells in renal allograft interstitial fibrosis

### 2.1 T cells: dual roles in fibrosis via inflammation and exhaustion

**Figure 1:**
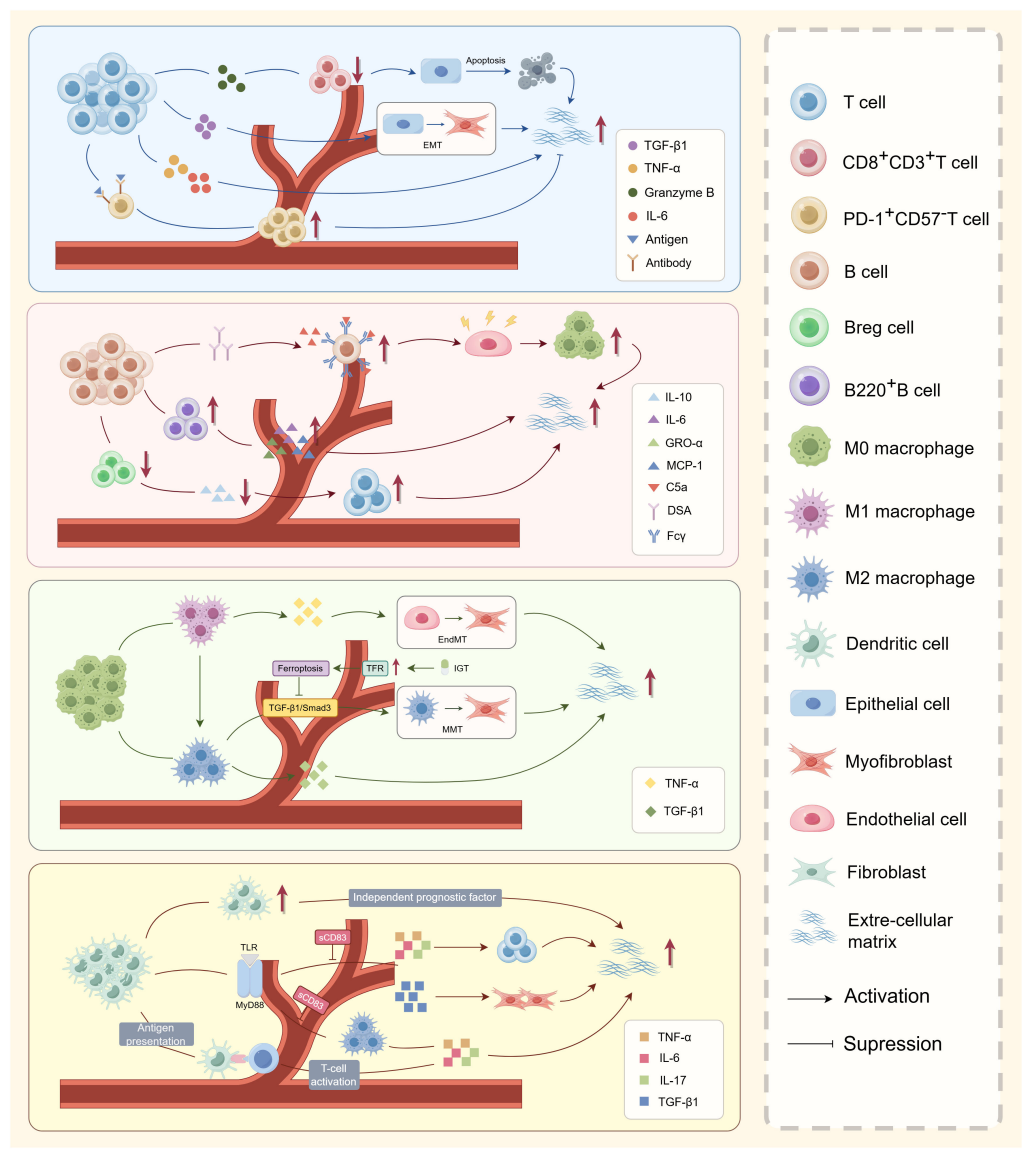
Role of immune cells in renal allograft interstitial fibrosis. T cells, B cells, macrophages, and dendritic cells orchestrate a complex immunoregulatory network that drives renal interstitial fibrosis during chronic allograft dysfunction. Activated CD8^+^ cytotoxic T cells and Th1/Th17 subsets promote fibrogenesis through secretion of proinflammatory cytokines (TNF-α, IL-6, and TGF-β), induction of EMT, and stimulation of fibroblast-to-myofibroblast transdifferentiation. Conversely, PD-1^+^ exhausted T cells exert antifibrotic effects by attenuating immune activation. B cells contribute via DSA-mediated endothelial injury and direct release of profibrotic mediators (e.g., GRO-α, MCP-1), while impaired Breg function exacerbates chronic inflammation. Macrophage polarization determines divergent fibrotic outcomes: M1 macrophages amplify inflammation through TNF-α and IL-1β secretion, whereas M2 macrophages undergo MMT under TGF-β1/Smad3 signaling, directly contributing to ECM deposition. DCs initiate adaptive immune activation via antigen presentation and secrete TNF-α, IL-6, and TGF-β, further enhancing fibroblast activation and ECM accumulation. Together, these immune cell subsets establish a self-reinforcing profibrotic microenvironment that underlies chronic renal allograft injury.

T cells are key mediators of adaptive immunity in renal transplantation, contributing to both acute rejection and chronic allograft injury through antigen recognition and proinflammatory cytokine release (**Figure 1**). Their role in renal interstitial fibrosis exhibits a functional duality, with some subsets driving fibrogenesis and others mitigating it via exhaustion-related mechanisms. From a profibrotic perspective, activated T cells contribute to interstitial fibrosis through both direct cytotoxic effects and indirect paracrine signaling. Wang et al. demonstrated that CD8^+^ cytotoxic T cells directly induce tubular epithelial cell apoptosis via the release of granzyme B and perforin in the unilateral ureteric obstruction (UUO) mouse model (19), while simultaneously activating the IL-6 and TGF-β signaling pathways, thereby initiating epithelial-mesenchymal transition (EMT) (20, 21). In addition, Th1 and Th17 subsets secrete proinflammatory and profibrotic cytokines, thereby sustaining chronic inflammation and amplifying fibrotic responses within the renal interstitium, ultimately exacerbating ECM deposition (22). In contrast, emerging evidence suggests that certain T-cell subsets may exert protective, anti-fibrotic effects. In a cohort study including 26 CTOT-01 recipients and 50 CTOT-19 kidney transplant recipients, Friburg et al. reported that chronic antigenic stimulation is associated with the development of an exhausted T-cell phenotype, which in turn correlates with reduced fibrosis severity. The exhausted T cells displayed high expression of inhibitory receptors, including programmed cell death protein 1 (PD-1) and T cell immunoglobulin and mucin domain 3 (TIM-3), and showed markedly impaired effector cytokine secretion (23). Although PD-1^+^CD57⁻ exhausted T cells lose conventional immune effector functions, they appear to exert protective roles through several interrelated mechanisms. Their diminished production of proinflammatory cytokines contributes to the alleviation of tubulointerstitial inflammation. Furthermore, activation of the PD-1/PD-L1 signaling pathway suppresses ongoing T-cell activation, thereby dampening immune-driven injury (24). Regulatory T cells (Tregs) also play a role in maintaining the exhausted phenotype of CD8^+^ T cells, further reinforcing an anti-inflammatory and anti-fibrotic microenvironment (25, 26). Thus, while activated CD8^+^ and Th1/Th17 cells promote fibrosis by mediating cytotoxic damage and sustaining inflammatory cytokine production, exhausted T cells are associated with reduced fibrotic progression due to their impaired effector function and immunomodulatory effects. These findings highlight the functional heterogeneity within T-cell populations and suggest that modulating T-cell exhaustion may represent a promising therapeutic strategy for limiting renal allograft fibrosis without broadly suppressing immune activity.

### 2.2 B cells: antibody-mediated injury and regulatory dysfunction

As a central component of the adaptive immune system, B cells contribute to renal allograft interstitial fibrosis through multiple pathogenic mechanisms in addition to their established role in AMR. These mechanisms include antibody-dependent injury, dysfunction of regulatory B-cell subsets, and the direct secretion of profibrotic mediators. In antibody-mediated injury, donor-specific antibodies (DSAs) produced by B cells activate complement cascades and Fcγ receptor-dependent signaling pathways. This activation induces endothelial damage and promotes macrophage recruitment, thereby triggering inflammatory responses that secondarily accelerate fibrotic progression (27). Regulatory B cells (Bregs) normally suppress T-cell activation by secreting anti-inflammatory cytokines such as interleukin-10 (IL-10), thereby limiting alloreactive immune responses. However, during chronic rejection, Breg function becomes progressively impaired. This dysfunction may lead to immune dysregulation and subsequent renal tissue injury, indirectly aggravating fibrotic remodeling (28). In addition, distinct B cell subsets directly produce chemokines and cytokines, thereby facilitating immune cell recruitment and contributing to fibrotic progression in the renal allograft (29). Consistent with these findings, Peng et al. reported a marked expansion of dominant B-cell receptor (BCR) clones, particularly those involving immunoglobulin lambda (IGL) chains, in patients with AMR, frequently preceding histological diagnosis. Notably, the persistence of these clones in some patients despite immunosuppressive therapy suggests ongoing subclinical B cell-driven injury that may silently fuel fibrotic remodeling over time (30). Collectively, these observations support the clinical utility of immune repertoire sequencing as a sensitive approach for monitoring B cell-mediated processes that contribute to chronic rejection and renal allograft fibrosis.

### 2.3 Macrophages: M1/M2 polarization and macrophage-to-myofibroblast transition

Macrophages, central innate immune cells derived from the mononuclear phagocyte lineage, exhibit remarkable heterogeneity and plasticity. Their polarization status critically influences inflammation, immune regulation, and fibrosis following kidney transplantation (31, 32). These cells originate either from embryonic tissue-resident populations or from circulating monocytes recruited during injury and inflammation (33). In the allograft microenvironment, monocyte infiltration is driven by hypoxia, necrosis, and tissue damage, with IFN-γ and LPS promoting differentiation into proinflammatory M1 macrophages, while IL-4, IL-10, and TGF-β favor M2 polarization (34). M2 macrophages further differentiate into subtypes (M2a, M2b, M2c, M2d) that variably regulate immune responses and tissue remodeling (35).

Functionally, M1 macrophages mediate acute rejection by secreting cytokines, activating NF-κB and JNK pathways, and aggravating tubulointerstitial injury (16, 36). After IRI, they can impair epithelial and endothelial regeneration and promote tubular atrophy (16). Persistent M1 dominance sustains chronic inflammation and promotes fibrosis. Notably, TNF-α from M1 cells facilitates endothelial-to-mesenchymal transition (EndMT), a process suppressed by TNF-α blockade (37). M2 macrophages, while anti-inflammatory and tissue reparative in acute injury, can paradoxically promote fibrosis under chronic conditions (38). Sustained TGF-β1 signaling activates Smad3-dependent macrophage-to-myofibroblast transition (MMT), generating α-SMA^+^ myofibroblasts from bone marrow–derived macrophages (16, 39, 40). In Smad3-deficient models, MMT is significantly reduced, confirming its regulatory role. Additional pathways, including the IL-4/NKT axis, PDGF, and MMP-9, are also involved in MMT and ECM remodeling (41–43). MMP-9 also facilitates EMT in tubular cells, and its deletion limits EMT-like phenotypes (43). Beyond soluble mediators, macrophage-derived exosomes enriched with miR-21a-5p were shown to activate fibroblasts via IL-6-inducible Notch2 signaling in a murine model of allogeneic kidney transplantation (44). Multi-omic analyses of chronic rejection specimens have revealed dense crosstalk among macrophages, myofibroblasts, and lymphocytes within ECM-rich niches, correlating with fibrotic progression (14, 45). Tubular epithelial cells and macrophages also form feedback loops: tubular cells secrete M-CSF to induce M2 polarization, which in turn supports epithelial survival and regeneration (46). Previous studies have shown that, in both clinical biopsy specimens from kidney transplant recipients and a bilateral renal IRI model using Syndecan-1–deficient mice, loss of protective components such as Syndecan-1 is associated with increased macrophage infiltration and more severe renal fibrosis (47). In summary, macrophages play dual and context-dependent roles in allograft fibrosis. M1 macrophages promote tissue damage through proinflammatory signaling, whereas M2 macrophages, despite their reparative profile, contribute directly to fibrogenesis via transdifferentiation. Therapeutic strategies that target polarization dynamics or interrupt MMT-related signaling pathways, particularly Smad3 or ferroptosis-related regulators, may offer effective antifibrotic interventions.

### 2.4 Dendritic cells: antigen presentation and profibrotic microenvironment modulation

Dendritic cells (DCs), the most potent professional antigen-presenting cells (APCs) of the innate immune system, play pivotal immunoregulatory roles in renal allograft fibrosis. Although the precise mechanisms underlying their direct profibrotic effects remain incompletely understood, accumulating evidence suggests that dendritic cells act as an independent prognostic factor for renal interstitial fibrosis. Increased DC infiltration is significantly associated with more severe fibrosis and poorer graft outcomes (48). Mechanistically, DCs may promote fibrogenesis through multiple pathways, including antigen presentation, T-cell activation, and remodeling of the inflammatory microenvironment. DCs possess distinct capacities for antigen capture, processing, and presentation. Through MHC class I and II molecules, DCs present donor alloantigens to CD8^+^ and CD4^+^ T cells, thereby activating alloreactive T-cell clones and initiating adaptive immune responses (49). This process not only mediates acute rejection but also contributes to chronic inflammation and fibrotic progression through persistent low-grade immune activation. Activated DCs secrete a broad spectrum of proinflammatory and profibrotic cytokines that directly modulate renal interstitial fibrosis (50–52). Notably, M. Ezzelarab et al. demonstrated that DCs activate effector T-cell subsets (Th1/Th17) via alloantigen presentation, triggering IFN-γ and IL-17 secretion that recruits and activates macrophages and fibroblasts, thereby establishing a profibrotic positive-feedback loop (53). DCs exhibit functional duality in renal allograft fibrosis: while acting as initiators of immune responses that activate alloreactive T cells, they also directly or indirectly facilitate fibrotic microenvironment formation through cytokine-mediated networks. Future studies should explore DC-targeted immunomodulatory strategies, including the induction of tolerogenic DCs or inhibition of key profibrotic mediators, to mitigate or reverse the progression of renal allograft fibrosis.

## 3 Role of epithelial cells in renal allograft interstitial fibrosis

### 3.1 Programmed cell death and ferroptosis in epithelial cells

**Figure 2:**
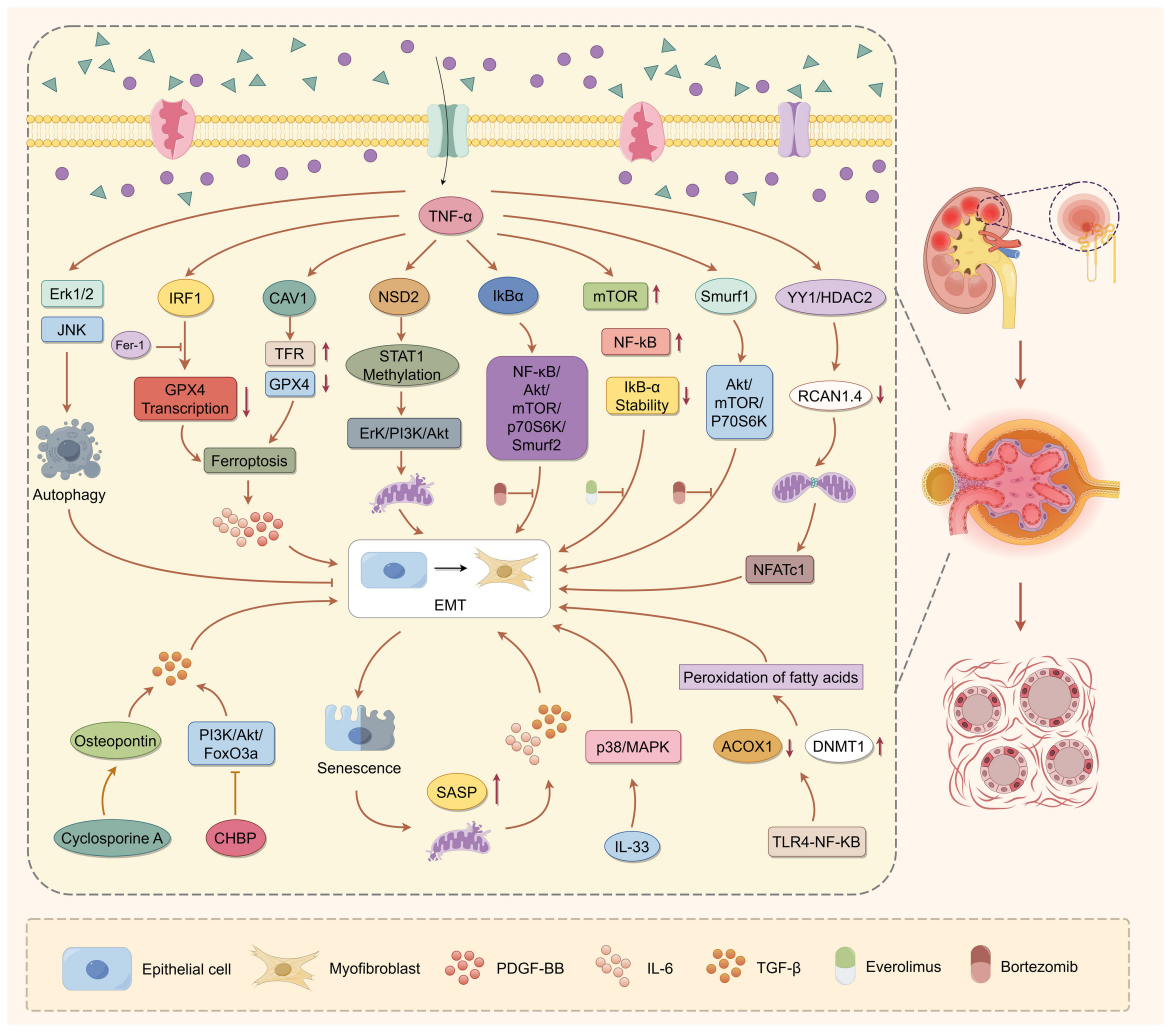
Role of renal tubular epithelial cells in allograft interstitial fibrosis. Renal tubular epithelial cells actively drive allograft interstitial fibrosis through ferroptosis and EMT. Inflammatory and metabolic stressors, including TNF-α and IRF1, suppress GPX4 expression, triggering ferroptosis and mitochondrial dysfunction. Ferroptotic RTECs release profibrotic mediators such as PDGF-BB and IL-6, which activate adjacent fibroblasts and enhance ECM deposition. Meanwhile, EMT is induced by inflammatory cytokines (e.g., TNF-α, IL-33), immunosuppressants (e.g., cyclosporine A), and metabolic disturbances via multiple signaling cascades—including NF-κB/Akt/mTOR/p70S6K/Smurf2, YY1/HDAC2/RCAN1.4, and NSD2/STAT1/Drp1. This transition is marked by the loss of epithelial markers, upregulation of mesenchymal proteins (α-SMA, fibronectin), and acquisition of migratory and secretory properties. Notably, ferroptosis and EMT form a feed-forward loop, wherein senescence, oxidative stress, and epigenetic dysregulation reinforce fibrotic signaling, accelerating progressive tissue remodeling in the renal allograft.

Renal tubular epithelial cells (RTECs), while essential for maintaining tubular function and homeostasis (54), also play an active role in the pathogenesis of interstitial fibrosis following transplantation. Their contribution is mediated primarily through programmed cell death (PCD) pathways and EMT (55) (**Figure 2**). Among the diverse forms of programmed cell death, apoptosis, necroptosis, pyroptosis, autophagy, and ferroptosis have all been implicated, with ferroptosis emerging as a central driver of epithelial injury and fibrotic progression. Mechanistically, ferroptosis in RTECs is initiated by intracellular iron overload and oxidative stress, leading to mitochondrial dysfunction and lipid peroxidation. In a *GPX4*^cys/-^ mouse model, Yang et al. demonstrated that renal ischemia-reperfusion injury (RIRI) disrupts [4Fe-4S] cluster-containing proteins such as lipoic acid synthase (LIAS), impairing protein lipoylation and triggering ferroptosis. This process also sensitizes cells to cuproptosis, highlighting a synergistic axis between iron and copper toxicity in early renal injury (56, 57). Ferroptotic RTECs contribute to fibrosis not merely through cell death but by actively modulating their microenvironment. These cells release extracellular vesicles (EVs) containing proinflammatory cytokines, damage-associated molecular patterns (DAMPs), and profibrotic microRNAs, which amplify fibroblast activation and immune infiltration (58). Additionally, senescent RTECs exacerbate ferroptosis through the senescence-associated secretory phenotype (SASP) and reactive oxygen species that reinforce a profibrotic milieu (54). Inflammatory signals such as TNF-α promote ferroptosis in renal tubular cells, partly by suppressing antioxidant defenses like GPX4 (59, 60). This leads to the release of profibrotic mediators, including IL-6 and PDGF-BB, which activate fibroblasts and enhance extracellular matrix deposition (61, 62). Taken together, ferroptosis represents a central link between epithelial injury, immune signaling, and fibrogenesis in renal allografts. Its integration with cellular senescence, EV-mediated signaling, and cytokine regulation identifies ferroptosis not only as a consequence of injury but also as a propagator of chronic fibrosis. These findings provide a mechanistic foundation for exploring ferroptosis-targeted therapies in the context of renal transplantation.

### 3.2 Epithelial-to-mesenchymal transition

EMT of RTECs is a critical pathological mechanism in renal interstitial fibrosis. This process is regulated by inflammatory signaling, mitochondrial dysfunction, epigenetic modifications, and metabolic reprogramming. Various injurious stimuli, including inflammatory cytokines, the immunosuppressant cyclosporine A, and ferroptosis, initiate EMT through distinct but interconnected pathways. Ferroptotic RTECs release proinflammatory cytokines to promote EMT in neighboring epithelial cells through paracrine signaling. This establishes a reinforcing “inflammation-EMT” feedback loop (56, 57). Additionally, EVs from injured TECs deliver fibrotic signals, including miRNAs and cytokines, contributing to EMT propagation (58). Senescent RTECs, characterized by mitochondrial dysfunction and acquisition of a SASP, release TGF-β and reactive oxygen species that directly link epithelial aging to fibrogenesis (54). Inflammatory signaling plays a central role in modulating EMT (63). TNF-α activates both the Akt/mTOR and NF-κB pathways, which upregulate Smurf2 expression and drive mesenchymal transformation (64). Pharmacological interventions that disrupt this cascade can mitigate EMT. For example, bortezomib stabilizes IκBα and suppresses NF-κB signaling, while everolimus induces autophagy and inhibits mTOR activity, collectively attenuating EMT and fibrosis (65, 66). Smurf1 also contributes to EMT by enhancing Akt/mTOR/p70S6K signaling through a positive feedback mechanism (67). Epigenetic regulation is another key contributor to EMT progression. YY1/HDAC2-driven RCAN1.4 suppression promotes mitochondrial stress and calcineurin/NFATc1 signaling, facilitating EMT (68). NSD2-mediated STAT1 methylation enhances mitochondrial fission and EMT via ERK/PI3K/Akt signaling (18). In parallel, metabolic dysregulation reinforces the EMT phenotype. Cyclosporine A promotes TEC migration and mesenchymal transformation by upregulating osteopontin (OPN) expression (69). Lipid metabolic disturbance, mediated by TLR4/NF-κB-induced DNMT1 upregulation and ACOX1 suppression, leads to lipid accumulation and oxidative stress, further accelerating EMT development (17). In summary, RTEC EMT arises from the convergence of inflammatory, metabolic, and epigenetic disruptions. Rather than acting in isolation, these pathways form a dynamic regulatory network that sustains fibrotic remodeling. Targeting critical nodes within this network, such as Smurf2, YY1/HDAC2, or metabolic regulators, may offer new therapeutic opportunities for renal fibrosis. Continued investigation is needed to clarify how these pathways interact to enable the development of precise antifibrotic strategies.

## 4 Role of stromal cells in renal allograft interstitial fibrosis

### 4.1 Endothelial cells: endothelial dysfunction and endothelial-to-mesenchymal

**Figure 3:**
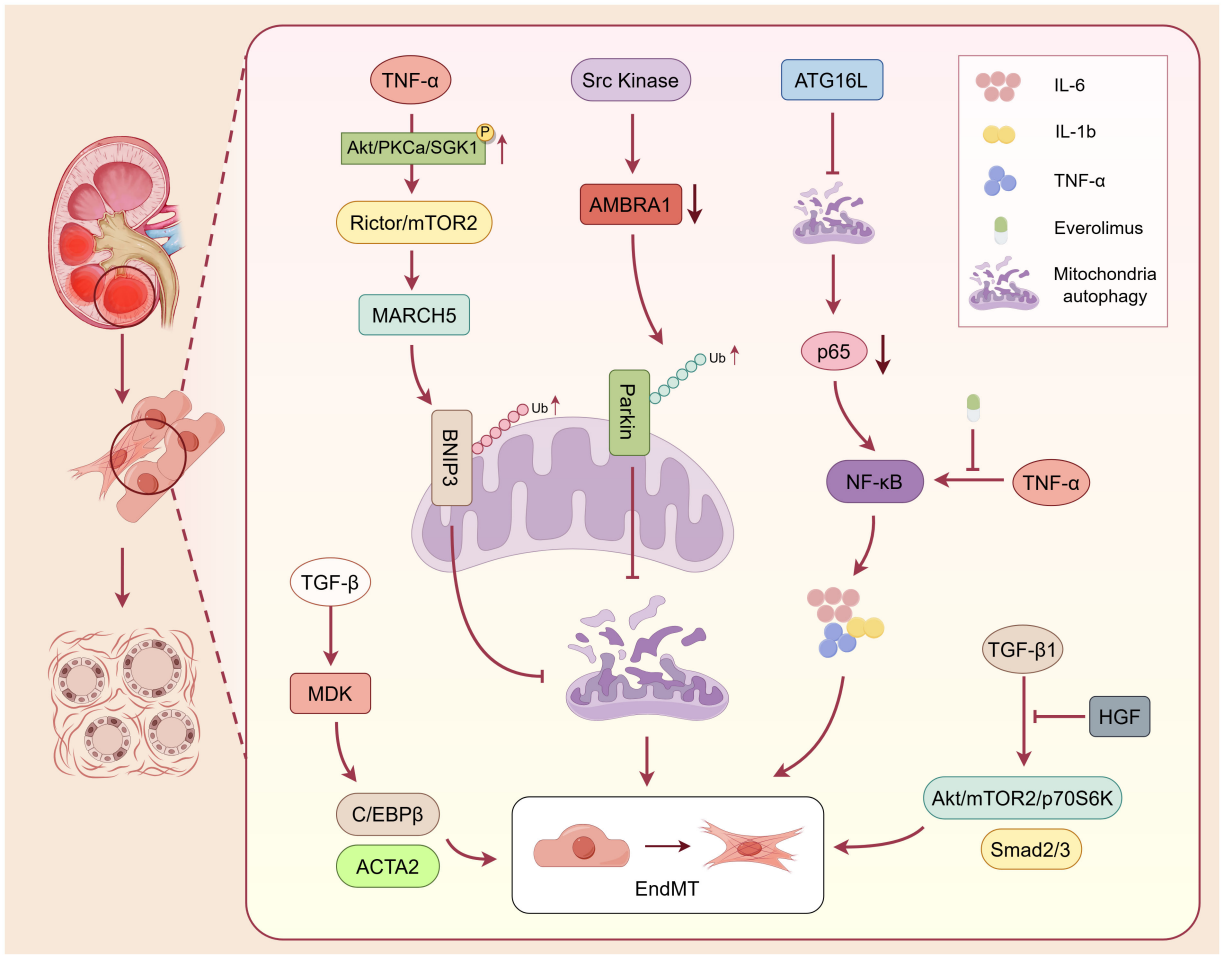
Renal vascular endothelial cells contribute to chronic allograft interstitial fibrosis through inflammatory injury, EndMT and impaired mitophagy. Endothelial cells promote chronic allograft interstitial fibrosis via sustained inflammatory activation, EndMT, and defective mitochondrial quality control. proinflammatory cytokines such as TNF-α, IL-1β, and IL-6 upregulate adhesion molecules (ICAM-1, VCAM-1, and P-selectin), facilitating leukocyte adhesion, capillary rarefaction, and microvascular dysfunction. Activated endothelial cells undergo EndMT through TGF-β1/Smad, NF-κB, and Akt/mTOR/p70S6K signaling pathways, exhibiting downregulation of endothelial markers (CD31, VE-cadherin) and increased expression of mesenchymal markers (α-SMA, collagen). MDK, upregulated by TGF-β, stabilizes the transcription factor C/EBPβ and enhances ACTA2 expression, further driving the EndMT process. Concurrently, impaired autophagy and mitophagy—mediated by NF-κB and Src/mTORC2 signaling—suppress AMBRA1- and BNIP3-dependent mitochondrial clearance, leading to accumulation of proinflammatory cytokines and mitochondrial dysfunction. Conversely, HGF inhibits TGF-β1–induced EndMT by attenuating Smad and mTOR signaling, exerting protective antifibrotic effects. Collectively, endothelial injury and phenotypic transition foster a proinflammatory and hypoxic microenvironment that perpetuates fibrogenesis, establishing endothelial cells as critical initiators and amplifiers of renal allograft interstitial fibrosis.

Renal vascular endothelial cells, specialized monolayer cells lining the vascular lumen, preserve renal function by regulating hemodynamics, maintaining filtration barrier integrity, and modulating immune and metabolic homeostasis (**Figure 3**). Emerging evidence indicates that capillary rarefaction, together with EndMT, jointly drives fibrotic remodeling underlying CKD progression (70). Wu et al. compared ICAM-1 knockout mice, CD18-deficient mice, and wild-type C57BL/6J mice using a lipopolysaccharide-induced sepsis-associated acute kidney injury model. They observed that that proinflammatory cytokines increase the expression of endothelial adhesion molecules, including ICAM-1, VCAM-1, and P-selectin on PTCs (71). This enhancement of endothelial-leukocyte interactions establishes a robust proinflammatory milieu, triggering endothelial apoptosis and consequent microcirculatory dysfunction. Furthermore, extensive capillary rarefaction aggravates endothelial dysfunction and inflammation. These changes promote endothelial apoptosis, EndMT, and disrupt the balance between pro- and anti-angiogenic factors (72). Converging evidence shows that inhibition of interleukin-1 receptor-associated kinase 4 (IRAK4) signaling attenuates renal interstitial cell proliferation and fibrosis, underscoring the pivotal role of proinflammatory cytokines in profibrotic signaling cascades (73).

The occurrence of EndMT represents an important mechanism in renal interstitial fibrosis, particularly in the setting of chronic allograft dysfunction. Yang et al. identified a subset of CD31^+^ACTA2^+^ endothelial cells in a partial EndMT state that serves as an important source of fibrotic cells in a murine unilateral ureteral obstruction model. In these cells, TGF-β induces high expression of *midkine* (*MDK*), which promotes EndMT by stabilizing the transcription factor C/EBPβ and upregulating ACTA2 expression. Knockout of *MDK* alleviates EndMT and attenuates fibrosis, suggesting that MDK may serve as a potential therapeutic target (74). Wang et al. revealed that TGF-β1 induces EndMT through activation of both the Akt/mTOR/p70S6K and Smad pathways in human umbilical vascular endothelial cells (HUVECs) (75). This process is characterized by increased expression of α-SMA and collagen, accompanied by downregulation of the endothelial marker CD31. Hepatocyte growth factor (HGF) antagonizes this process by inhibiting TGF-β1/Smad and Akt/mTOR/p70S6K signaling, thereby exerting antifibrotic effects (76). Beyond TGF-β1, TNF-α also promotes EndMT through NF-κB activation, a process that can be suppressed by the mTOR inhibitor everolimus (66). Autophagy impairment likewise contributes to EndMT modulation. In a chronic renal allograft dysfunction rat model, ATG16L downregulation reduced autophagic flux, limited p65 degradation, and consequently enhanced NF-κB signaling, accompanied by increased release of proinflammatory cytokines (66). Gui et al. further demonstrated that Src kinase activation downregulates AMBRA1, promoting Parkin ubiquitination and degradation, thereby impairing mitophagy and elevating IL-6 levels to induce EndMT (77). In addition, Feng et al. showed that activation of Rictor/mTORC2 signaling promotes phosphorylation of Akt, PKCα, and SGK1, upregulates MARCH5 expression, and mediates BNIP3 ubiquitination and degradation, thereby suppressing mitophagy and exacerbating EndMT in a murine allogeneic kidney transplantation model (78). Overall, renal vascular endothelial dysfunction contributes to the multifaceted regulatory network of EndMT. This contribution is mediated by proinflammatory factor-driven microvascular injury and EMT.

### 4.2 Fibroblasts and myofibroblasts: activation and differentiation

Fibroblasts, the most abundant stromal cell population within the renal interstitium, are primarily responsible for synthesizing and maintaining the ECM under physiological conditions. In the context of renal allograft rejection, fibroblasts undergo significant phenotypic and functional changes, including activation, proliferation, and transdifferentiation into myofibroblasts. This process is central to the development and progression of renal allograft interstitial fibrosis. Activated fibroblasts contribute to fibrogenesis through multiple profibrotic signaling pathways, including those mediated by TGF-β1, Wnt/β-catenin, and JAK/STAT6 axes (79, 80). Myofibroblast differentiation is marked by the *de novo* expression of α-SMA, acquisition of contractile stress fibers, and enhanced synthesis of ECM components such as collagen I and fibronectin (81). In the setting of allograft injury, ferroptotic epithelial cells release inflammatory mediators, including platelet-derived growth factor-BB (PDGF-BB) and IL-6. These factors act in a paracrine manner to activate interstitial fibroblasts and promote their transition into myofibroblasts, thereby exacerbating fibrotic remodeling (59). Recent studies have identified key transcriptional and epigenetic regulators of fibroblast activation in the context of renal allograft fibrosis. Zhao et al. revealed that the MRTF-A-ZEB1-IRF9 regulatory axis drives fibroblast-myofibroblast transition by suppressing anti-fibrotic IRF9, and deletion of MRTF-A effectively attenuates fibrosis in murine models (82). Likewise, NOX4-dependent reactive oxygen species (ROS) signaling has been shown to synergize with TGF-β1 to maintain the myofibroblastic phenotype and matrix production (81). In the allograft microenvironment, immune-stromal crosstalk plays a crucial role in fibroblast dynamics. Notably, using an *in vivo* UUO model combined with CCR7 deficiency and CCL21 blockade, Wada et al. demonstrated that CCR7^+^ fibroblasts are recruited to allogeneic grafts via SLC/CCL21, promoting ectopic lymphoid structure formation and local fibrosis (83). Additionally, Th2 cytokines have been shown to stimulate bone marrow-derived fibroblast precursors through JAK3/STAT6 signaling, further contributing to myofibroblast accumulation during chronic rejection (80). Although significant advances have been made in understanding fibroblast activation and myofibroblast differentiation in renal fibrosis, their precise roles in the context of renal allograft remain unclear. The unique immune environment of the transplanted kidney, influenced by alloimmune injury, immunosuppression, and donor-specific factors, may shape fibroblast behavior in ways that differ from native kidney fibrosis. The heterogeneity of fibroblast subpopulations, including differences in origin, activation thresholds, and profibrotic potential, is increasingly recognized as a key determinant of fibrosis progression. Elucidating how these cells interact with immune infiltrates and injured epithelial cells will be essential for identifying specific targets for anti-fibrotic therapy. With the application of single-cell transcriptomics (scRNA-seq) and spatial profiling, future studies are expected to reveal novel fibroblast subsets that contribute to chronic allograft injury and represent new avenues for intervention.

### 4.3 Pericytes: pericyte-to-myofibroblast transition and capillary rarefaction

Renal pericytes are specialized perivascular cells that ensheath glomerular and peritubular capillaries, playing critical roles in vascular stability, ECM homeostasis, and intercellular signaling within the renal microenvironment (84). In the setting of renal allograft injury, increasing evidence implicates pericytes as key contributors to interstitial fibrosis through their transition into myofibroblasts. Kramann et al. demonstrated that, upon injury, pericytes detach from the vascular wall and undergo pericyte-to-myofibroblast transition (PMT), thereby promoting renal fibrogenesis (85). PMT plays a central role in renal interstitial fibrosis and is regulated by multiple profibrotic signaling pathways. These pathways include sustained activation of TGF-β and PDGF signaling, as well as the TLR4/CD36 axis coupled with FUT8-mediated core fucosylation and mitochondrial apoptosis. In addition, TGF-β1-induced PI3K-Akt-mTOR signaling drives metabolic reprogramming, such as HKII upregulation and enhanced glycolysis, while lipopolysaccharide-binding protein (LBP) can activate the TLR4/TGF-β pathway under septic conditions (86–90). In the context of renal transplantation, pericyte activation and dysfunction may be further exacerbated by alloimmune responses. Xu et al. reported that autophagy impairment in pericytes exacerbates vascular instability and promotes fibrotic remodeling, compromising long-term graft survival in a murine AMR kidney transplantation model (91). Additionally, capillary rarefaction, a hallmark of chronic allograft dysfunction, is closely linked to pericyte loss. As pericytes detach and differentiate into matrix-producing myofibroblasts, capillary network integrity declines. This leads to reduced oxygen delivery and aggravated hypoxia, which in turn perpetuates fibrosis through HIF-mediated pathways (92). Despite growing recognition of their importance, the regulatory circuits underlying PMT and its interactions with the microenvironment, including endothelial cells, immune infiltrates, and metabolic stressors, remain incompletely characterized. Targeting PMT or promoting pericyte stabilization represents a promising therapeutic strategy not only to limit myofibroblast expansion but also to preserve microvascular integrity in renal allografts.

## 5 Multicellular crosstalk in renal allograft interstitial fibrosis

**Figure 4:**
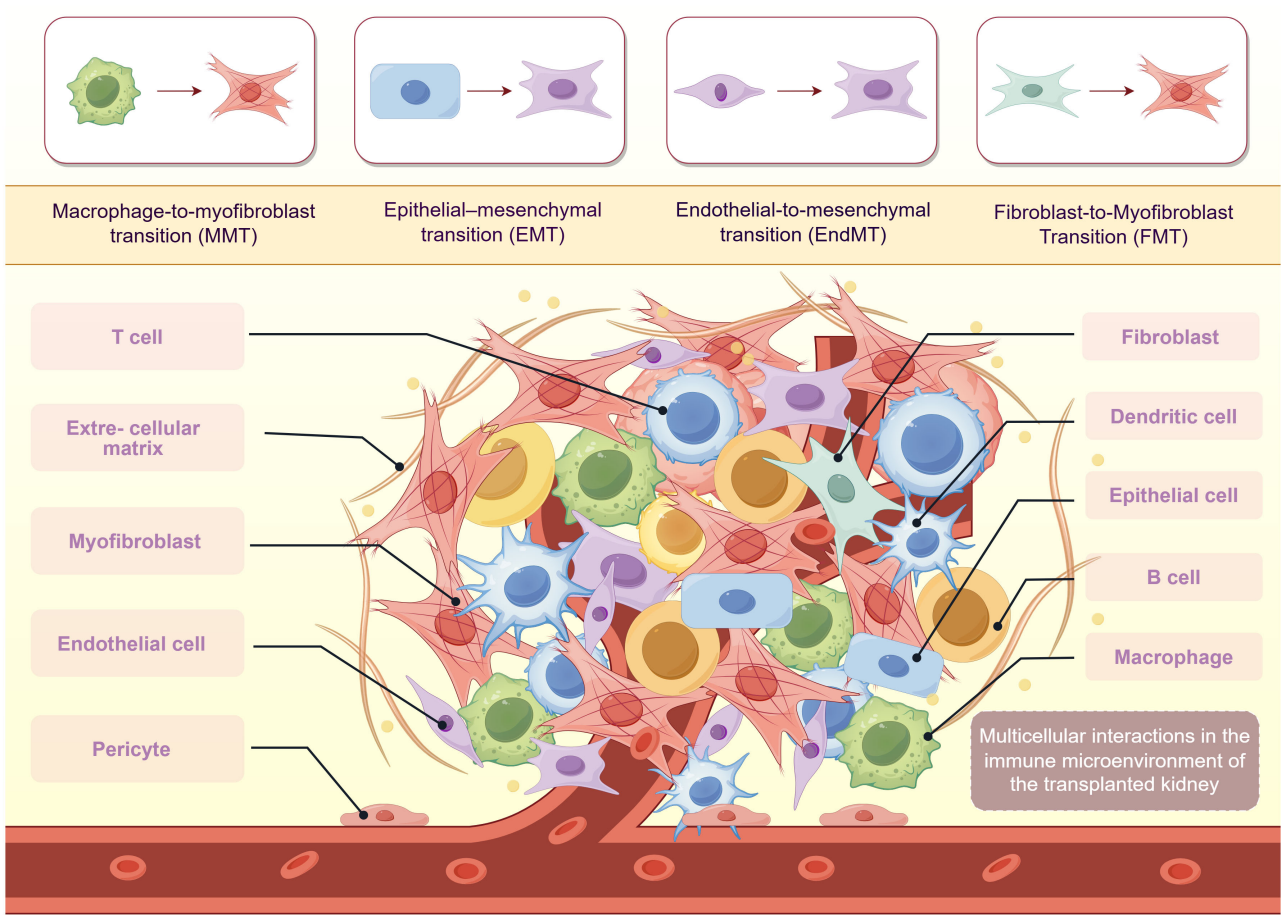
Immune–nonimmune cellular crosstalk in renal allograft interstitial fibrosis. Reciprocal interactions between immune and non-immune cells sustain chronic fibrotic progression. Cytokines and chemokines (e.g., TNF-α, IL-6, TGF-β, MCP-1) released by T cells, B cells, macrophages, and dendritic cells activate epithelial, endothelial, and stromal cells, inducing ferroptosis, EMT, EndMT, and fibroblast activation. In turn, injured epithelial and endothelial cells amplify inflammation by recruiting and polarizing macrophages, forming a self-perpetuating inflammatory–fibrotic loop. This multicellular crosstalk establishes a persistent profibrotic microenvironment driving chronic allograft dysfunction.

Renal allograft fibrosis arises from complex interactions among tubular epithelial cells, immune cells, stromal fibroblasts, and vascular endothelial cells. These cell types communicate through cytokines, extracellular vesicles, and metabolic signals, forming a pathological network that sustains inflammation and matrix deposition (**Figure 4**). RTECs, when exposed to stress such as ferroptosis or senescence, release proinflammatory mediators including IL-6 and TGF-β. These signals activate surrounding immune cells, particularly macrophages and T cells (54, 56, 57). CD8^+^ and Th17 T cells exacerbate epithelial injury through cytotoxic mechanisms and inflammatory cytokines (22), while M1 macrophages contribute to tissue damage and EMT by secreting TNF-α and IL-1β (16, 36). Endothelial cells also actively participate in fibrotic remodeling. Inflammatory cytokines released by macrophages and T cells induce EndMT, a process by which endothelial cells acquire myofibroblast-like properties and contribute directly to extracellular matrix deposition (37). EndMT also leads to capillary rarefaction, impairing local perfusion and further exacerbating epithelial hypoxia and injury. TEC-derived TGF-β and oxidative stress products can reinforce this process, establishing cross-talk between the tubular and vascular compartments. EV signaling plays a central role in cell–cell communication. TECs undergoing ferroptosis or senescence release EVs carrying DAMPs, miRNAs, and fibrotic cytokines, which activate fibroblasts and attract macrophages (58). Macrophages also contribute to the fibroblast pool through MMT (39, 40). Once activated, fibroblasts secrete ECM and profibrotic mediators that feedback on epithelial and endothelial cells, reinforcing mesenchymal transformation. Recent transcriptomic analyses of chronic rejection have revealed spatial proximity and intercellular signaling between lymphocytes, myofibroblasts, and endothelial cells within fibrotic niches (14, 45). These multicellular feedback loops maintain a self-sustaining fibrotic microenvironment. Intervening in these circuits by targeting EV release, TGF-β signaling, or the mesenchymal transition of epithelial and endothelial cells may offer promising strategies for antifibrotic therapy.

## 6 Multi-omics dissection of interstitial fibrosis and tubular atrophy in kidney allografts

IF/TA represent the predominant pathological features contributing to late allograft failure in kidney transplantation. The underlying molecular mechanisms are highly complex, involving disruptions in immune regulation, cellular metabolism, and tissue repair processes (14). Conventional monitoring parameters such as serum creatinine and proteinuria are often delayed indicators and lack specificity. While allograft biopsy remains the diagnostic gold standard, it is inherently invasive and carries procedural risks (93). Consequently, the application of multi-omics technologies has gained increasing attention for deciphering the molecular landscape of IF/TA and developing noninvasive biomarkers to improve long-term graft outcomes (94, 95).

### 6.1 Bulk transcriptomics

Bulk transcriptomics is a platform that enables comprehensive gene expression profiling of entire kidney allograft biopsy specimens (96). Early studies have demonstrated that transcriptomic signatures associated with inflammation and extracellular matrix remodeling correlate with the histological severity of IF/TA and predict long-term graft outcomes. These findings suggest that specific gene modules are mechanistically involved in the pathogenesis of chronic allograft injury (97–99). More recently, bulk RNA sequencing of renal tissue or alternative biological samples such as urine has provided new perspectives on the molecular mechanisms of IF/TA. This approach has enabled the identification of early diagnostic biomarkers and predictors of disease progression (100). For instance, Lubetzky et al. developed a urine cell mRNA profiling technique that quantifies absolute levels of specific transcripts. This method allows for the detection of T cell–mediated rejection, antibody-mediated rejection, IF/TA, and BK virus nephropathy from urine samples (101). In addition to biomarker discovery, bulk transcriptomics has facilitated mechanistic insights through functional enrichment analysis. Multiple studies have shown that IF/TA is associated with the activation of immune dysregulation and fibrotic pathways (100, 102). Choi et al. validated the involvement of specific signaling cascades, including PI3K-AKT, JAK-STAT, and TGF-β/Smad pathways, supporting these observations. One of the major strengths of bulk transcriptomic analysis is its capacity to leverage large cohorts for identifying gene expression patterns with prognostic significance (103). However, a key limitation of this approach is the inability to assign gene expression profiles to specific cell types. Therefore, bulk transcriptomic data often serve as a foundational resource for subsequent single-cell and spatial transcriptomic investigations to delineate the cellular origins of broad molecular patterns.

### 6.2 Single-cell and single-nucleus transcriptomics

scRNA-seq is a powerful technique that profiles the transcriptome of individual cells by enzymatically dissociating fresh tissue, thereby enabling high-resolution analysis of tissue and cellular heterogeneity. In contrast, single-nucleus RNA sequencing (snRNA-seq) performs RNA profiling on isolated nuclei, serving as a viable alternative for frozen or difficult-to-dissociate samples. This approach mitigates dissociation-induced transcriptional artifacts and preserves cell type–specific transcriptomic information (104). Recent studies have demonstrated that snRNA-seq enables systematic profiling of fibrotic kidney allografts, overcoming limitations posed by archived or frozen specimens. This technique has elucidated the transcriptional states and functional characteristics of various immune cells and resident kidney parenchymal cells involved in chronic injury (105, 106). Notably, work by Kuppe and colleagues identified distinct subpopulations of fibroblasts, monocytes/macrophages, and tubular epithelial cells, each exhibiting unique functional properties during fibrogenesis. These included profibrotic fibroblast subsets, mononuclear phagocytes sustaining chronic inflammation, and epithelial cells displaying dedifferentiated or stress-related phenotypes (107). Cell-level resolution further revealed that fibroblasts, immune cells, and epithelial cells collaborate yet diverge in their roles in matrix deposition, inflammation amplification, and tissue remodeling. These findings enabled precise attribution of fibrosis- and inflammation-related pathways previously observed in bulk transcriptomic studies to their corresponding cellular sources (107, 108). Additionally, Shetty et al. reported that proximal tubular epithelial cells undergo phenotypic conversion into a mixed injured tubular (MT1) state. These cells coexpress markers of activated fibroblasts and myofibroblasts, secrete transient extracellular matrix components, and recruit inflammatory cells, thereby serving as major drivers of fibrotic progression (14). Together, single-cell and single-nucleus transcriptomics offer transformative tools for delineating the cellular basis of interstitial fibrosis and tubular atrophy in kidney allografts. They also provide critical insights into cell–cell interaction networks and inform the development of targeted interventions against specific pathological cell subsets.

### 6.3 Spatial transcriptomics

Although single-cell transcriptomic technologies enable high-resolution dissection of cellular heterogeneity, they inherently lose spatial information due to tissue dissociation. This loss limits the ability to interpret transcriptional data within the architectural and microenvironmental context of the tissue (109). Spatial transcriptomics overcomes this limitation by preserving tissue morphology while enabling *in situ* gene expression profiling. This allows researchers to map molecular signatures to specific anatomical regions and histopathological structures, providing spatial context to transcriptional alterations (109, 110). In studies of chronic kidney injury and fibrosis, spatial transcriptomics has revealed that immune-related genes and extracellular matrix remodeling pathways exhibit distinct spatial expression patterns. In particular, chemokine networks and inflammatory signaling pathways show differential localization between areas of interstitial expansion and adjacent intact parenchyma (111). These regionalized transcriptional features suggest that the local immune–matrix microenvironment plays a pivotal role in orchestrating chronic fibrogenesis. Although the application of spatial transcriptomics to kidney transplant biopsies remains limited, its potential is considerable. This technology holds promise for uncovering cell-type heterogeneity and immune landscape dynamics within IF/TA lesions, thereby advancing our understanding of the molecular and cellular mechanisms underlying rejection and chronic fibrosis (112).

### 6.4 Proteomics

Proteomics is the large-scale, systematic study of the entire protein complement encoded by a genome under specific biological conditions. By directly profiling protein abundance and post-translational modifications, proteomics complements transcriptomic findings and offers a more functional perspective on cellular processes (113). In kidney transplantation, Mortensen and colleagues conducted deep quantitative proteomic analyses on allograft biopsy specimens and identified key proteins that strongly correlate with the degree of interstitial fibrosis. These include coagulation factor XIII A chain, uridine phosphorylase 1, and actin-related protein 2/3 complex subunit 2 (114). Additionally, mass spectrometry–based quantitative proteomic studies have been applied to body fluids, such as urine, and have revealed multiple proteins associated with IF/TA (115, 116). Beyond biomarker discovery, proteomics has enhanced mechanistic insights into IF/TA (117). In the context of antibody-mediated rejection, Tataranni et al. reported that RTECs express IL-17, potentially mediated by complement activation and JAK2 phosphorylation. This sustained inflammatory activation contributes to chronic fibrotic remodeling (118). Collectively, proteomics provides functional validation of transcriptome-derived findings and offers a complementary layer of insight into the molecular mechanisms underlying chronic allograft injury. It also supports the development of noninvasive monitoring strategies by enabling protein-based biomarker discovery.

## 7 Therapeutic strategies in renal allograft interstitial fibrosis

Therapeutic strategies for renal allograft fibrosis aim to slow the progression of fibrosis, preserve renal function, and prolong graft survival. It has been reported that approximately two-thirds of fibrotic lesions emerge within the first year after transplantation, with the extent of interstitial fibrosis exceeding that of tubular atrophy (119). At present, research specifically targeting renal allograft fibrosis remains limited, and severe established fibrosis is currently almost irreversible. Therefore, in the quest to manage chronic interstitial fibrosis of the transplanted kidney, early and effective interventions directed at distinct cellular targets following transplantation are crucial for halting or reversing fibrotic progression. Recent studies further suggest that such interventions may be categorized into immune-targeted and non-immune-targeted strategies, with a focus on modulating key cellular interactions and signaling pathways (**Table 1**).

**Table 1:** Current and emerging therapeutic strategies for renal allograft interstitial fibrosis.

Target cell/process	Representative strategy/drug	Mechanism of action	Clinical evidence	KDIGO 2025 status
T cells	Low-dose IL-2/IL-2 muteins	Promotes Treg expansion; modulates PD-1^+^ exhausted T cell responses	Phase I (NCT02411230)	Under investigation; Not included
B cells	Rituximab, Belatacept, Atacicept	Depletes CD20^+^ B cells; blocks co-stimulation (CD80/86); BAFF inhibition	Approved for AMR; Atacicept in Phase II (NCT01949166)	Approved for AMR; Not specific to fibrosis
Macrophages	CSF-1R inhibitors, CCR2 antagonists	Modulate M1/M2 polarization; inhibit recruitment and MMT	Preclinical; early trials for CCR2 (e.g., CCX872)	Not included
Dendritic cells	TREM-1 inhibitors; 2-deoxyglucose (2-DG)	Suppress DC activation and glycolytic reprogramming	Preclinical evidence in animal models	Not included
Ferroptosis in epithelial cells	GPX4 activators, miR-20a-5p mimics	Inhibit ferroptosis in tubular cells; reduce inflammatory/fibrotic signaling	Preclinical renal transplant models	Not included
EMT	Everolimus, Bortezomib, HGF, SnoN upregulation	Inhibit NF-κB/Akt/mTOR/Smurf2; restore epithelial integrity	Everolimus approved; Bortezomib in trial phase	Everolimus recommended in KDIGO 2025
EndMT	TGF-β receptor inhibitors (e.g., Galunisertib), Notch inhibitors (e.g., DAPT)	Block TGF-β/Smad and Notch signaling during endothelial transition	Preclinical and early phase studies	Preclinical; Not included
Myofibroblasts	CAR-T/CAR-M against FAP^+^ cells	Deplete activated myofibroblasts selectively	Preclinical pulmonary/cardiac fibrosis models	Preclinical; Not included
Pericytes	PDGF inhibitors, TLR4 inhibitors	Prevent pericyte detachment and PMT	No clinical trials yet; preclinical in renal models	Not included
Cross-target: EV/mRNA therapies	Engineered EVs, mRNA-HGF, IL-10	Reprogram fibrotic microenvironment; deliver miRNAs/anti-fibrotic proteins	Liver/kidney models; mRNA in Phase I trials (e.g., Moderna)	Experimental stage; Not included

### 7.1 Immune-targeted therapies: engineering immune cells for anti-fibrotic precision

Immune cells play a dominant role in the positive feedback loop between chronic inflammation and fibrotic matrix deposition (120, 121). Among them, CD8^+^ T cells enter a state of functional exhaustion under persistent antigenic stimulation, characterized by the upregulation of inhibitory receptors such as PD-1 and TIGIT, accompanied by reduced secretion of cytokines including IL-2 and IFN-γ. The PD-1/PD-L1 signaling pathway plays a pivotal role in modulating CD8^+^ T cell function during chronic kidney injury, and its blockade has been explored as a potential strategy to mitigate renal fibrosis (122, 123). In addition, Tregs are crucial for maintaining immune tolerance; administration of low-dose IL-2 has been reported to promote Treg expansion and reinforce an anti-fibrotic immune milieu (123, 124). Notably, IL-2 muteins and anti-IL-2 complexes have been developed to selectively expand Tregs while avoiding effector T cell activation, representing an immune-balancing approach with reduced toxicity (125). Myeloid-derived suppressor cells (MDSCs) have also emerged as potent immunoregulatory cells that mitigate transplant rejection and fibrosis. They function by suppressing effector T-cell responses, inducing Tregs, inhibiting dendritic and NK cell activation, and reprogramming macrophages toward anti-inflammatory phenotypes via IL-10, arginase-1, and iNOS signaling (126, 127). B cells contribute to transplantation-associated fibrosis through three major mechanisms: AMR, cytokine secretion and antigen presentation (30). Anti-CD20 monoclonal antibodies such as rituximab have been widely used in the management of AMR, while combination therapy with agents like belatacept or BAFF inhibitors (e.g., atacicept) holds promise for mitigating B cell–mediated nonspecific inflammatory responses (128). Liang et al. found that immune repertoire sequencing enables early identification of dominant BCR clones (especially IGL chains) in AMR, often before histological detection. Persistent clonal expansions despite immunosuppression suggest ongoing subclinical B-cell–driven injury, and immune profiling may serve as a tool for both prediction and therapeutic monitoring of fibrotic progression (129). Macrophages, on the other hand, determine the fibrotic fate through their polarization states. Classically activated M1 macrophages amplify proinflammatory signaling, whereas alternatively activated M2 macrophages, despite their role in tissue repair, also promote myofibroblast differentiation via the secretion of TGF-β1 and Arg1. Recent studies have demonstrated that CSF-1R inhibitors, Kv1.3 channel blockers, and CCR2 antagonists can precisely modulate macrophage polarization at different stages of fibrosis, thereby suppressing ECM deposition (130). DCs, as key antigen-presenting cells, are influenced by the TREM1 signaling pathway and glucose metabolic reprogramming. Hyperactivation of DCs amplifies local inflammation and triggers excessive T cell activation, forming a pathogenic feedback loop. Animal studies have shown that blockade of TREM1 signaling or the use of glycolytic inhibitors such as 2-deoxy-D-glucose (2-DG) can attenuate DC activation and effectively prevent immune-driven fibrogenesis (131).

Cell-engineering-based immunotherapies, particularly chimeric antigen receptor (CAR)-modified immune cells, are increasingly recognized as transformative approaches for the treatment of fibrotic diseases. CAR-T cells targeting fibroblast activation protein (FAP) have demonstrated potent efficacy in selectively depleting activated myofibroblasts, a major ECM-producing population, in preclinical models of cardiac and pulmonary fibrosis, where ECM remodeling disrupts normal tissue architecture (132, 133). These findings build upon the understanding that fibrosis involves not only cellular hyperplasia but also profound dysregulation of ECM dynamics, orchestrated by interactions among immune cells, fibroblasts, and endothelial cells. Although CAR-T therapies have yet to be applied in renal allograft fibrosis, the successful targeting of FAP^+^ myofibroblasts in cardiac tissue suggests translational feasibility across organ systems where similar stromal components drive chronic fibrotic progression (10). Furthermore, CAR-macrophages (CAR-M) combine innate phagocytosis with engineered plasticity and have shown encouraging preclinical results in tissue remodeling and fibrotic resolution (134). The combination of immune checkpoint inhibitors with CAR platforms may unlock synergistic avenues for controlling chronic inflammation while targeting the fibrotic stroma.

### 7.2 Non-immune targeted therapies: reprogramming tissue microenvironments and metabolism

Non-immune cellular populations such as tubular epithelial cells, endothelial cells, fibroblasts, and pericytes are central to the progression of fibrosis via phenotypic transitions and microenvironmental remodeling. RTECs contribute to fibrosis through EMT, ferroptosis, and defective autophagy. Ferroptosis, an iron-dependent form of regulated cell death, has been identified as a critical mechanism in IRI, primarily affecting RTECs (56). Targeting key ferroptosis regulators such as ACSL4 and GPX4 significantly reduces interstitial collagen deposition and slows fibrotic progression (135, 136). Notably, miR-20a-5p has been shown to alleviate tubular injury by suppressing ACSL4-dependent ferroptosis, highlighting its potential as a therapeutic small RNA in post-transplant fibrosis (137, 138). Enhancing autophagic flux in tubular cells also offers antifibrotic benefits. For instance, everolimus promotes autophagy and inhibits EMT by modulating inflammatory signaling pathways (66). EMT driven by TGF-β1 can also be suppressed by HGF-induced SnoN upregulation or proteasome inhibition (e.g., bortezomib), both of which exhibit efficacy in experimental models (65, 139). Endothelial cell dysfunction represents an early and pivotal event in post-transplant fibrosis. Studies have demonstrated that targeting endothelial-associated pathways, including TGF-β receptor inhibitors (e.g., galunisertib), VEGF-reconstituting agents, and EndMT blockers such as the Notch inhibitor DAPT, can yield significant antifibrotic effects (140, 141). Endothelial-protective agents such as Angiopoietin-1 mimetics, antioxidants (e.g., NAC), and S1P receptor agonists further support vascular stability and reduce rarefaction-induced hypoxia (142). Spatial transcriptomic analyses have recently revealed specific proinflammatory and metabolically impaired endothelial subpopulations within fibrotic niches, paving the way for precise subtype-targeted therapy (143). Fibroblasts, as terminal ECM-producing effectors, are activated by TGF-β signaling and metabolic reprogramming. Inhibiting lactate production via dichloroacetate (DCA) or enhancing fatty acid oxidation may downregulate myofibroblast activation (144). Similarly, epigenetic modulation, such as targeting NSD2 or HDAC complexes, offers new strategies to reverse fibrotic programming.

Recent advances in mRNA nanotechnology and engineered EVs have introduced innovative platforms for targeted antifibrotic therapy. mRNA therapeutics enable transient, non-integrative, and cell-specific expression of regenerative or regulatory proteins, thereby circumventing risks associated with gene editing or protein instability. In preclinical models of liver fibrosis, lipid nanoparticle-delivered mRNA encoding HGF or hepatocyte nuclear factor 4 alpha (HNF4A) significantly attenuated collagen deposition and restored hepatic architecture by antagonizing TGF-β signaling and promoting epithelial regeneration (145–148). Dual-mRNA delivery of HGF and IL-10 further enhanced therapeutic outcomes by synergistically modulating fibrogenic and inflammatory pathways (146). Meanwhile, EVs engineered to deliver antifibrotic nucleic acids, such as anti-miR-21 or siRNA against TGF-β receptors, have demonstrated efficacy in reprogramming fibrotic microenvironments across organ systems, with minimal immunogenicity and high cell-targeting precision (58, 147). Similarly, Yang et al. developed RTEC-targeting engineered EVs carrying siRNA against TGF-β receptor I, which suppressed downstream Smad2/3 signaling and significantly reduced fibrosis in kidney transplant models. This siRNA-EV platform exhibited enhanced specificity, reduced off-target effects, and durable antifibrotic efficacy, offering a safe and adaptable nucleic acid delivery system for renal applications (127). These nucleic acid-based therapies are further empowered by AI-integrated multi-omics analytics, which enable early identification of fibrosis-prone cell subsets and matching of therapeutic targets. Collectively, these modular platforms hold promise for organ-adaptable, programmable, and precision-guided antifibrotic interventions, including future applications in renal transplantation.

## 8 Conclusions and future perspectives

Renal allograft interstitial fibrosis represents the defining pathological feature of CAD and remains the primary barrier to long-term transplant survival. This review underscores the triadic interplay among immune, epithelial, and stromal cell populations as the central axis orchestrating fibrotic progression in the transplanted kidney. Within this fibrotic network, immune cells such as Th1/Th17 lymphocytes, M1/M2-polarized macrophages, and dendritic cells initiate and amplify fibrogenesis through sustained production of inflammatory and profibrotic mediators. Simultaneously, epithelial cells undergo maladaptive transitions to contribute to paracrine signaling and myofibroblast recruitment. Stromal components, including endothelial cells, fibroblasts, and pericytes, not only respond to these cues through EndMT, FMT and PMT but also form feed-forward loops that perpetuate extracellular matrix deposition and microvascular rarefaction. In summary, this triad of cellular miscommunication, compounded by metabolic dysregulation and epigenetic reprogramming, establishes a dynamic and self-reinforcing fibrotic microenvironment. Importantly, recent findings highlight protective immune subsets and molecular targets as promising entry points for therapeutic intervention. The success of agents such as everolimus and proteasome inhibitors illustrates the feasibility of targeting key nodal pathways within this multicellular network.

Future research should move beyond linear pathway-based interpretations and adopt a spatially and transcriptionally resolved framework to understand renal allograft fibrosis. At the basic research level, integrating scRNA-seq with spatial transcriptomics will allow for the high-resolution dissection of fibrotic tissue architecture. Building upon this, we propose the concept of “fibrotic microenvironment classification”, which seeks to categorize distinct fibrotic niches based on the spatial distribution of dominant cell types, signaling nodes, metabolic programs, and immune states. This multidimensional classification system may provide a novel framework for identifying spatially defined therapeutic vulnerabilities and constructing precision stratification models for renal transplant recipients. At the computational level, artificial intelligence (AI) can be leveraged to integrate clinical phenotypes, molecular omics, and imaging features to generate predictive models of fibrosis progression and therapeutic responsiveness. These models will support early diagnosis and dynamic risk monitoring across the transplant continuum. At the translational level, therapeutic innovations such as engineered extracellular vesicles, mRNA-based reprogramming agents, and cell state–specific inhibitors targeting EMT, EndMT, MMT, FMT and PMT may enable tailored intervention across distinct fibrotic states identified by the microenvironmental classification. At the clinical level, embedding multiparametric, stage-specific fibrosis classification into clinical workflows will require coordinated efforts in biomarker validation, adaptive immunosuppressive strategy design, and multicenter clinical trial deployment. While the KDIGO 2025 guidelines acknowledge the antifibrotic potential of agents such as mTOR inhibitors and B cell-targeted therapies, many novel approaches involving immune regulation, epithelial plasticity, and stromal cell intervention have yet to be fully incorporated. To bridge this gap, future efforts must focus on integrated multi-omics research, the development of AI-supported clinical models, and the design of clinical trials aligned with fibrosis stage and cellular context. A comprehensive systems biology framework that integrates immune, epithelial, and stromal interactions, guided by spatial and transcriptional profiling of the fibrotic microenvironment, has the potential to advance precision therapy and improve long-term outcomes for renal allograft recipients.
